# Care and justice reasoning in nurses’ everyday ethics

**DOI:** 10.1177/09697330241312379

**Published:** 2025-01-05

**Authors:** Soile Juujärvi, Birgitta Tetri

**Affiliations:** 3254 Laurea University of Applied Sciences; 3254 Laurea University of Applied Sciences; University of Helsinki

**Keywords:** Care reasoning, ethical dilemmas, ethics of care/care ethics, justice reasoning, practical nurses

## Abstract

**Background:** The ethics of care and justice represent two modes of moral reasoning that nurses use in solving real-life ethical dilemmas.

**Research aim:** The present study investigated what types of dilemmas nurses encounter in everyday work and to what extent they use care versus justice reasoning to solve them.

**Research design:** The study used a cross-sectional survey design. Participants reported a real-life ethical dilemma and its resolution through an online survey. Open-ended data were analysed with an adjusted taxonomy of real-life moral dilemmas and moral orientations. Quantified data were analysed with statistical methods (χ^2^-test, analysis of variance).

**Participants and research context:** Participants were 334 registered nurses and 177 practical nurses from four health and social care organisations in Finland.

**Ethical considerations:** The study was approved by the Research Ethics Committee of Finnish Institute for Health and Welfare.

**Findings:** Nurses reported six types of ethical dilemmas. Nurses used more care reasoning on the needs of others and conflicting demands dilemmas than on applying rules, social pressure and reacting to transgression dilemmas. Applying rules and needs of others dilemmas were the most common types of ethical dilemmas in both occupations. Practical nurses reported more non-ethical dilemmas than registered nurses did.

**Discussion:** Ethical dilemmas of nurses are diverse, and the use of care and justice reasoning is differentiated according to the type of dilemma. Care reasoning dominates nurses’ ethical decision-making when responding to the needs of patients. Nurses use justice reasoning when they apply regimens, rules and procedures. In everyday ethics, care and justice reasoning complement each other.

**Conclusions:** Highly regulated healthcare environments prefer rule-oriented justice reasoning that may supersede care reasoning in addressing patients’ situations. Focus on technical-professional expertise may further hamper nurses’ ethical decision-making. Nursing education and management should encourage nurses to use ethical concepts and values in their work.

## Introduction

A care-based ethic^1,2^ is fundamental for quality nursing practice that is based on nurses’ moral commitment to build caring relationships with patients and respond appropriately to their specific needs.^3^ While the concept ‘the ethic of care’ has several meanings and philosophical roots within nursing,^4^ psychologist Carol Gilligan^5^ was the one that argued for its distinctive quality in ethical decision-making processes. According to her, the ethics of care and justice constitute different modes of moral reasoning that may elicit different ethical decisions. While the ethic of justice emphasises upholding role-related duties and commitments, standards, rules, and principles as the primary moral criteria for ethical decision-making, the ethic of care emphasises promoting the welfare of others, preventing harm, or relieving burdens, hurt and suffering as such.^2,5^ In ethical deliberation, care reasoning builds on the representation of concrete situations as entirely as possible, whereas justice reasoning aims to find a generalisable solution that can be applied to all similar cases.^6^

Research in moral psychology has established that the ethic of care and justice represent distinctive modes of moral reasoning that perceive, interpret, and solve moral dilemmas in different ways.^7^ The main factor explaining the choice of moral reasoning is a type of dilemma rather than gender – women preferring the ethic of care and men the ethic of justice – as Gilligan originally proposed.^8^ More specifically, ethical dilemmas relating to selfish temptations and transgressions invoke predominantly justice reasoning, whereas ethical dilemmas relating to the needs and expectations of others invoke predominantly care reasoning.^1,9^ Consequently, several studies have shown that nursing students^10,11^ and nurses in different organisational contexts use both modes of moral reasoning.^12–15^ It has also been evidenced that the ethics of care and justice perspectives are integrated with each other in nurses’ mature moral thoughts. This means that at the highest levels of moral reasoning, professionals’ ethical decisions are derived from shared ethical values and principles, such as patient well-being and human dignity.^7,16^ Studies furthermore show that ethical dilemmas nurses encounter in work are diverse including team and organisation level issues beyond patient-related issues.^17–19^ It is not yet known to what extent nurses use care and justice reasoning when facing and solving different kinds of ethical dilemmas. The present study aims to respond to this research gap.

To illuminate the research gap further, the ethic of care has a somewhat contradictory status in the field of nursing. It has been valorised as a recommendable attitude and virtue for nurses that increases their ethical sensitivity and responsibility,^3,20,21^ but still regarded inferior to principle-based theories in ethical decision-making processes.^4^ One explanation might be that justice-based ethical principles are learned through education, whereas care concepts can be derived from the common language of nurses solving everyday moral issues.^22^ Previous research shows that the nurses’ ethical decision-making processes are complex, involving multiple personal and contextual factors.^23^ In justifying their decisions, nurses may rely on medical and nursing knowledge, personal values and experiences, ethical theories, and principles^23,24^ or they can consult colleagues or supervisors.^25^

This study adds the perspectives of care and justice reasoning to ethical decision-making. They have been established as generic modes of moral thought^26,27^ and serve as cognitive bedrocks for professional ethical concepts and provide alternative and complementary frameworks for moral reasoning.^28,29^ Various normative ethical theories can be linked to individuals’ moral reasoning through these frameworks. Virtue and feminist ethics, as well as several nursing theories, can be seen anchored in the ethic of care,^4^ whereas deontological and utilitarian theories are originated from the ethic of justice.^29^ The ethics of care and justice thus do not explain exhaustively nurses’ nuanced ethical decision-making but present a necessary cognitive bottom-line for real-life moral reasoning.

Ethical dilemmas have traditionally been defined as opposing values or alternatives with unwelcome consequences and a choice must be made between them.^26,30^ Given the diversity of ethical issues nurses face, this definition is too narrow for research purposes. Ethical concerns do not always require immediate choice but may, for example, involve moral appraisals on the quality of care or organisational restraints.^17,31^ Following our methodology,^2^ we define an ethical dilemma broadly as a situation at work in which one feels unsure of what is the morally right thing to do.

### Objectives

The objectives of the present study were to investigate (1) what types of ethical dilemma nurses encounter in everyday work, and (2) to what extent they use care and justice reasoning in solving different types of ethical dilemma. Because our sample consists of registered and practical nurses, (3) we control for differences between these occupations. In addition, (4) we provide brief descriptions for each type of dilemma to illuminate further quantitative findings.

## Materials and methods

### Context of the study

The present study was a part of a research project that explored the transition in health and social services in Finland and its impact on employment, competence needs and development. The project was conducted in four forerunner areas where municipalities had joined to reorganise public services in line with the forthcoming national health care and social services reform that was realised in 2023. In the reform, responsibility for organising services was transferred from municipalities to more significant self-governing regions called wellbeing counties. Primary healthcare and social services were integrated to ensure better care and treatment especially for people who are frequent users of services.^32^ At the time of data-gathering, workers in forerunner areas already worked in new organisational units and different forms of service integration accompanied with multi-professional work were enhanced.^33^ Notably, these target organisations did not include private health and social care organisations.

Our target group included practical and registered nurses in four region-wide organisations in southern and eastern Finland. They comprise the largest and second-largest groups of healthcare workers in Finland.^34^ Nursing education takes place at universities of applied sciences, takes 3.5 years, and complies with the European Union’s regulations. In 2020, 83% of registered nurses worked in public services. One of the recent developments is the establishment of advanced practical nursing roles that are missing official nationwide regulations except for nurse prescribing.^35^

Practical nurse is a nationally regulated vocational qualification in social and health care based on an upper secondary degree. Three-year education gives wide-ranging basic capabilities, specialised competence and vocational skills in various areas of social and health care. Practical nurses are expected to carry out work following the regulations, operating practices, values, and occupational ethics of their field.^36^ Ethical guidelines for practical nurses define human dignity, self-determination, justice, equality, responsibility, and community as basic ethical principles.^37^ Practical nurses work extensively in public primary and specialised medical care and social care, including homecare and residential care.^36^

### Data collection and ethical considerations

The data were collected by an online survey that comprised 59 questions on changes in work and organisations, leadership, competence needs, and work-related wellbeing, including background variables and two open-ended questions on ethical dilemmas, which were utilised in this study. The research plan was approved by the Research Ethics Committee of Finnish Institute for Health and Welfare (decision 2017:3, 757). Participants received written information about the study through e-mails. They were informed that participation was voluntary, and the anonymity of participants was ensured.

The total sample was drawn from the personnel registers of four public organisations providing health care and social services in the region. Contact persons in organisations delivered online surveys targeted to all workers with permanent jobs in the spring and autumn of 2017, followed by two reminders. Because the survey was delivered online through email lists, the exact number of participants who actually received it could not be tracked. The calculated response rate based on the personnel registers for practical nurses was 15% (*n* = 333) and for registered nurses, 22% (*n* = 559), respectively. The final sample included 177 practical nurses (13%) and 344 registered nurses (18%) who had responded to two open-ended questions on ethical dilemmas and its resolution.

Respondents were asked to describe an ethical dilemma with the following instructions: Go back in your mind to an event or situation from the recent past in your work in which you were unsure about how to act in order to do the right thing. Describe the problem. What aspects caused the problem for you and why? How did you act in that event or situation? The question was followed by the second question: How do you know whether you acted rightly or wrongly? Briefly give reasons for your answer.^2^

### Analysis

Reported dilemmas were classified using the adapted taxonomy of real-life dilemmas,^1^ which involved categories as follows: Prosocial dilemmas related to others’ welfare involve reacting to the *needs of others* and *conflicting demands*. In the needs of others dilemma, one feels unsure whether they are responsible for engaging in some proactive behaviour on another person’s behalf and what their duties or responsibilities towards the person in question are. In the *conflicting demands* dilemma, one is faced with two or several people making inconsistent demands on them, often with implications for their relationship, and the respondent must decide whose expectations to fulfil. Antisocial dilemmas involve reacting to selfish temptations and transgressions. In the *temptation* dilemma, one is faced with the temptation to meet one’s needs or desires or advance personal gain by behaving dishonestly, immorally, unfairly or ungratefully. In the *transgression* dilemma, a decision must be made regarding what to do about a transgression, injustice, crime, or violation of rules that the respondent has witnessed.^1^ The *social pressure* dilemma forms the third main category. Therein, the respondent feels implicitly or explicitly pressured by another person or group to engage in identity-inconsistent behaviours that violate their values or moral identity.^1^

In addition to the categories mentioned above, recent studies^38^ have documented two additional types relevant to working life. The *applying rule* dilemma refers to a situation where a respondent is unsure about how a specific rule, instruction, order, command, or law should be applied or followed. In contrast, the *internal conflict* dilemma refers to a situation where a respondent feels conflicted due to the discrepancy of situational demands and personal psychological resources or contradictory internal values. The conflict is internal because others do not necessarily observe it.^38^

The second author classified ethical dilemmas reported by participants and checked by the first senior author. Some dilemmas could be classified into two or several categories, and a decision was made as to which category seemed to be the primary one for a participant. Furthermore, twenty-eight per cent of reported dilemmas (*n* = 144) described work-related problems that did not include normative statements and could not be scored in terms of moral reasoning. Based on the content, these non-ethical dilemmas were further classified into knowledge-based problems (*n* = 46), practical problems (*n* = 40), familiarization (*n* = 20), and resource issues (*n* = 17).

The data was then analysed and scored using the coding scheme for care and justice orientations.^2^ Considerations about maintaining relationships, promotion of the welfare of others, preventing harm or relieving burdens, hurt or suffering, and practical consequences of ethical choice were rated as care. Considerations about role-related obligations, duties and commitments, maintaining standards, rules, values and moral principles, and reasoned justifications of ethical choice were rated as justice. Considerations of care and justice were summed up for each respondent. Following Gilligan & Attanucci,^39^ a five-point scale was used to assess respondents’ relative use of care and justice reasoning, varying from 0 (exclusively justice-oriented) to 100 (exclusively care-oriented). Thus, the final score represents the percentage of care reasoning that is inversely related to the percentage of justice reasoning.

The analysis was conducted by the second author and checked by the first author, who had prior expertise in scoring. Disagreements were resolved through discussion. The analysis was made as blind as possible to the classification of dilemmas in order to avoid biased scoring. Data were analysed through descriptive statistics, chi-square tests, and analysis of variance that were conducted using SPSS Statistics 26 software.

## Findings

### Background variables

The mean age for practical nurses was 47 years (*SD* = 11.8), and for registered nurses, 43.4 years (*SD* = 10.1), respectively. Practical nurses had worked in the healthcare field for an average of 15.5 years (*SD* = 11.3), while registered nurses had an average of 12.4 years of experience (*SD* = 9.3). 88% of both practical nurses and registered nurses were female. Age and gender were representative of nurse populations in Finland, except for the mean age of practical nurses: 47 years compared with 42.8 years in the population.^40^

The participants’ work spanned various settings, highlighting the increasingly diverse and fragmented nature of nursing tasks across different care environments. Among registered nurses, one-third (33%, *n* = 136) were employed in primary healthcare, while 54% (*n* = 220) worked in specialised healthcare. In contrast, 39% (*n* = 85) of practical nurses worked in primary healthcare, and 15% (*n* = 33) were in specialised healthcare. In home care, only 4% (*n* = 17) of registered nurses were involved in this sector, compared to 27% (*n* = 59) of practical nurses.

### Type of dilemma and occupation

Table 1 displays the distribution of participants’ ethical dilemmas according to occupation. Both occupational groups reported dilemmas (problems) that could not be classified according to the current taxonomy. These non-ethical dilemmas accounted for 40% of practical nurses (*n* = 70) and 22% of registered nurses (*n* = 74). The association between occupational group and type of dilemma was significant, *χ*^2^ (6, *N* = 521) = 22.62, *p* < .001. Compared to registered nurses, practical nurses reported more non-ethical dilemmas and complementarily less applying rules and needs of other dilemmas as a whole.

**Table 1.** Type of ethical dilemma, occupation, and care reasoning.

Type of dilemma	Registered nurses		Practical nurses		Total		Care reasoning^a^	
	n	%	n	%	N	%	Mean	SD
Needs of others	77	29	28	26	105	28	65.24_a_	31.87
Conflicting demands	17	6	10	9	27	7	58.33_a,b_	37.34
Social pressure	29	11	15	14	44	12	27.84_c_	30.60
Transgression	39	14	19	18	58	15	29.31_c_	31.11
Applying rule	90	33	31	29	121	32	28.10_c_	29.15
Internal conflict	18	7	4	4	22	6	45.45_b_	35.04
Total	270	100	107	100	377	100	41.78	35.38

Values with different subindices (a,b,c) differ from each other at level *p* < .05.

^a^Mean percentage of justice reasoning is inversed to mean percentage of care reasoning.

Among registered and practical nurses, the most common ethical dilemmas were applying the rule (33% and 29%), followed by the needs of others (29% and 26%). Both occupational groups reported other types of dilemmas less frequently: 14% and 18% for transgression, 11% and 14% for social pressure, 6% and 9% for conflicting demands, and 5% and 6% for internal conflict. None of the participants reported the temptation dilemma included in the taxonomy of real-life dilemmas.^1^

### Moral reasoning and type of dilemma

Needs of other dilemmas invoked highest care reasoning, whereas applying rule dilemmas invoked lowest care reasoning and, complementarily, highest justice reasoning (see Table 1). The analysis of variance on care reasoning showed significant main effects for the type of dilemma, *F* (5, 365) = 25, *F* (5, 365) = 25.42 and for occupational group *F* (1, 365) = 4,02, *p* <. 001, as well as significant interaction effects, *F* (5, 365) = 3.36, *p* < .01

Post-hoc comparisons between types of dilemmas with Duncan’s procedure revealed that needs of others, conflicting demands and internal conflict dilemmas invoked higher care reasoning than social pressure, transgression, and applying rules dilemmas, *p*s < .05. In addition, needs of others dilemmas invoked higher care reasoning than internal conflict dilemmas, *p* < .05. In other words, needs of others and conflicting demands dilemmas were more care-focused, whereas social pressure, transgression, and applying rules dilemmas were more justice-focused, internal conflict dilemmas ranked in the middle.

In general, practical nurses used higher care reasoning (*M* = 47.90, *SD* = 39.58) than registered nurses (*M* = 51.10, *SD* = 34.66) who complementarily used higher justice reasoning. Paired comparisons explicated that practical nurses (*M* = 87.50, *SD* = 19.84) used higher care reasoning than registered nurses (*M* = 57.14, *SD* = 31,64) on needs of others dilemma, *t* (−5,84, *df* = 76,72) = −5.84, *p* < .001, meaning that registered nurses used therein higher justice reasoning than practical nurses.

### Description of ethical dilemmas

#### Needs of others

Registered nurses focused on assessing particular care needs to ensure patients’ well-being or benefit. They had to find the best solution for the patient’s situation or choose among treatment lines or places. The responsibility for patient care may have already officially been transferred to another unit or person, but the nurse felt obliged to complete the task and ensure the continuity of care and patient safety. Registered nurses, in particular, often felt that their new responsibilities for care assessment surpassed limits of their professional expertise. Practical nurses’ work involved hands-on care, and they had to often consider the well-being of patients who resisted or disagreed about care actions. The scarcity of staff caused risks to patient safety and feelings of frustration among nurses. Poor information flows across organisational units often compromised the excellent care nurses tried to provide.

#### Conflicting demands

Dilemmas represented situations where patients, colleagues, and family members directed different needs, wishes, or expectations to nurses who needed to reconcile contradictory views while ensuring patient safety and continuity of care. Practical nurses, working mainly in elderly care, often encountered demands from family members that contradicted organisations’ practices. Disagreements for both registered and practical nurses concerned mainly care-related issues: treatment lines, drug prescriptions, rehabilitation activities, and choice of care placement. Unclear instructions and procedures promoted the emergence of differences of opinion. Insufficient staff resources caused situations where the demands of the organisation and the needs of clients did not meet.

#### Social pressure

Nurses felt pressured by patients, family members, colleagues, or members of multi-professional teams to act in ways that could violate their nursing values by compromising good care. Patients pressured to get urgent doctor’s appointments or wanted to have a dose of medication deviating from the physician’s prescription. Family members demanded unrealistic rehabilitation activities for patients or changes in treatment, or they opposed discharge. Physicians may require nurses to perform tasks for which they do not have authority or legal permission. For example, the physician instructed nurses that they should not send patients to the emergency department ‘too easily’. Nurses typically persisted with their moral convictions and did not yield to social pressure in these situations.

#### Transgression

On transgression dilemmas, nurses witnessed or perceived unethical or illegal behaviour of patients or colleagues and felt obliged to intervene in situations in appropriate ways. Patients could be violent or abusive, and nurses had to decide how to promptly respond to them. Colleague-related dilemmas were far more mixed. Nurses witnessed when colleagues neglected their duties, made medication errors, or mistreated patients, and they had to intervene in situations or report them to supervisors or organisations to ensure patient safety. Colleagues or other team members broke the rules, bullied others, or did not comply with agreed procedures in work units, causing harm to other nurses or patients. Supervisors treated the staff poorly or unfairly. Nurses solved these situations in mixed ways. They need to consider whether laws and rules were violated and who was morally or legally responsible for acting, and they often justified their interventions by the importance of restoring victims’ welfare.

#### Applying rule

Unclear, inadequate, or contradictory rules and procedures complicated both registered and practical nurses’ work. Registered nurses’ dilemmas concerned regimens, medication, discharge procedures and operating models, and they did not know how to apply them in the best ways. Unclear rules and procedures were especially present when nurses worked as substitutes in foreign wards. In difficult situations, they might ask for advice from a supervisor or colleague, which could nevertheless cause a delay in care. When supervisors were out of reach, nurses had to make decisions alone. When available, multidisciplinary teams or colleagues were consulted, and advice was sought to ensure the correct course of action. Unclear or strict rules caused extreme difficulties for practical nurses who were responsible for holistic basic care but did not have the authority to make care decisions necessary to ensure patients’ well-being.

#### Internal conflict

Nurses had doubts about their professional competence, skills, and integrity when encountering new situations and demanding tasks, and their self-confidence was further hampered by poor induction procedures and a lack of help and support. Nurses felt that their professional inadequacy may risk patient safety. As a solution, nurses may refuse to do tasks assigned to them or ask for help from supervisors and colleagues. Internal conflicts were mainly reported by newly graduated nurses.

#### Non-ethical dilemmas

Non-ethical dilemmas involved practical, knowledge-based, or resource-related issues. Respondents were bothered by plain prudential concerns of the dilemma and did not mention any moral aspect, such as consequences for people’s welfare or one’s professional integrity. These challenges often stemmed from unfamiliarity with protocols, insufficient resources, or inadequate training. For example, staff struggled with operational discrepancies or unclear guidance, which led to improper task execution or communication breakdowns. Practical mistakes, such as using suboptimal equipment due to time constraints or delivering incomplete injections, highlighted the need for better procedures and logistical support.

## Discussion

The present study aimed to investigate what types of ethical dilemmas registered and practical nurses encounter in everyday work and to what extent they use care and justice reasoning in solving different kinds of ethical dilemmas. Findings emphasise the diversity of ethical dilemmas nurses face in their complex work environments and explicate the differentiated roles of care and justice for their everyday ethics. Dilemmas varied from patient care to workplace and organisational issues, covering six types of ethical dilemmas. Prosocial and applying rule dilemmas both comprised roughly one-third of ethical dilemmas, featuring the recent landscape of nurses’ everyday ethics. Needs of others and conflicting demands invoked predominantly care reasoning and applying rule dilemmas justice reasoning, respectively. These findings illustrate tensions between ethically sound patient care and organisational demands in highly regulated healthcare systems.^17,23^

The needs of other dilemmas mainly concerned nurses’ needs assessment and concrete caregiving tasks that were a legitimate part of their job descriptions. These dilemmas can be considered prototypical nursing dilemmas because they reflect a caring attitude and responsiveness to patients’ needs in their particular situations.^3,4^ Ethically sound care is based on reciprocal relationships, empathic understanding and nuanced knowledge gathering on patients’ features and contexts^3,13,41^ Nurses felt morally responsible for delivering such care within organisational and professional limits. Ethical concerns arose, especially when nurses were not able to ensure whether patients’ needs were appropriately met due to limited time or restricted roles within care pathways. Findings furthermore suggest that practical nurses were able to focus more on basic care, whereas registered nurses need to consider more rules and procedures framing situations. Consequently, registered nurses might be less focused on patients’ wellbeing and become inattentive to their holistic needs when they prioritise executing regimens and organisational instructions. Registered nurses also felt ethically impaired when they encountered demanding tasks, especially in advanced role positions. In Finland, the tasks of physicians have been shifted to nurses in varied ways, bringing them new responsibilities and competence demands; for example, nurses have independent appointments and limited prescription rights in primary health care.^35^ Findings indicate that such autonomous roles can bring excessive moral distress for nurses unless properly handled through instruction and professional guidance.

Complementarily, applying rule dilemmas informed about nurses’ moral obligations to follow and uphold rules and procedures concerning patient care at multiple levels. The magnitude of these dilemmas may be due to the highly regulated nursing practice but may also reflect nurses’ need to conform to established rules and practices. Dierckx de Casterle et al.^16^ have pointed out that nurses are inclined to conventional moral reasoning that is characterised by pursuits to maintain and apply uniformly existing norms, laws and hierarchies to ensure social order.^29^ It thus differs from post-conventional reasoning that draws on principles of good care in ethical liberation and takes a critical stand against harmful or unjust conventions.^7,16^ Guidelines, rules, and procedures provide a necessary framework for nursing practice, but they should serve as a means to promote patient well-being rather than being ends in themselves.^16^

Conflicting demands and social pressure dilemmas formed almost one-fifth of ethical dilemmas, representing an important minority. These dilemmas are hard to solve because nurses need to deal with the expectations of other people, and their decisions might have consequences for relationships.^42^ Contradictory views originated from different opinions and values or poor information. It is worth noting that nurses in the present study did not feel compelled to please actors but rather acted as intermediaries of conflicted views. In turn, social pressure dilemmas were often linked to nurses’ positions as gatekeepers of access to care or medication. In resolving these conflicts, nurses combined perspectives of care and justice ethics by enhancing the principle of patient well-being as the ultimate moral criterion. These findings evidence nurses’ experiences navigating in disagreement with family members and other professionals, especially physicians.^43^ While unequal relationships between nurses and physicians have previously been regarded as an important source of moral distress,^30,44^ present findings signal relatively equal relationships allowing nurses to exercise their moral agency in multi-professional encounters. This is consistent with recent observations of nurses’ autonomous roles in Finland.^35^

The important unexpected finding was that a substantial number of reported dilemmas cannot be classified as ethical dilemmas because they include neither normative statements referring to the welfare of others (the ethic of care) nor moral principles, such as rules, norms, laws, and values (the ethic of justice). It is worth noting that any ethical principle raised by participants could have been classified as a justice-based consideration, and therefore, the absence of ethical concepts was a major cause of non-ethical dilemmas. There are several interlinked explanations for their disproportionately high number in the sample. First, the questionnaire was lengthy and questions on ethical dilemmas were placed at the end of the questionnaire. Due to time pressure and fatigue, participants may have read the instructions poorly and written superficial responses. With more elaboration, those dilemmas might have turned into ethical dilemmas. Second, participants may have recognised morally distressing situations, such as a lack of resources or poor staff familiarisation but have not been capable of articulating ethical concerns due to a lack of appropriate language. Third, participants did not recognise ethical issues in their environments at all. According to our understanding, non-ethical dilemmas may partly be due to the aforementioned inadequacies in the methodology, but they also inform about growingly complex and challenging healthcare environments requiring both technical and ethical competencies.^17,23^

### Strengths and limitations

There are some methodological concerns. The most significant limitation of this study is a low response rate and time-consuming questionnaire that further decreased the number of respondents and might have deteriorated the quality of open-ended responses. Consequently, respondents might have more positive attitudes towards ethical issues than non-respondents, and therefore findings are positively biased. Classification of dilemmas into single categories was somewhat artificial because many dilemmas included several ethical aspects. In addition, the data were collected before the COVID-19 pandemic, which might have altered nursing practices. On the other hand, the size of the sample remained considerable, including both registered and practical nurses that represent a remarkable share of health care workforce. Findings provide a comprehensive overview of nurses’ ethical dilemmas and decision-making in the ongoing change of healthcare organisations.

## Conclusions

The ethics of care and justice play essential roles in nurses’ everyday ethics. Nurse-patient encounters are grounded in the ethic of care and are simultaneously regulated by regimens, rules, and procedures that can enhance or restrict ethical actions. Therefore, both aspects should be critically evaluated in reflective ethical decision-making. Standardised nursing regimens derived from evidence-based practice^41^ and operating models striving for economic efficiency^45^ overlook the importance of reciprocal nurse-patient relationships and, therefore, require ethical reflection when implemented in practice. The ethic of care as a process involves responding to patients’ and nurses’ evaluations of how caring needs are met.^41,43^ Organisational care arrangements should enable reciprocity even in brief encounters to ensure ethically sound care. Otherwise, highly regulated healthcare environments may supersede care reasoning in addressing patients’ situations impoverishing the quality of nursing. This study has also implications for nursing education. A sole focus on technical-professional expertise may hamper nurses’ ethical decision-making capacities. Therefore, education should embrace different parts of the ethical decision-making process, involving the ability to recognise ethical issues, problem-solving capacities, prioritising ethical values over other values, and implementation of decisions.^29,45^ Nursing education and management should encourage nurses to articulate ethical aspects of their work through ethical concepts such as values because otherwise, it is difficult to recognise, raise, and discuss ethical issues in the workplace. This is especially important for practical nurses who work in the vicinity of patients, addressing their daily needs. New technologies and operational models entail new ethical dilemmas, and therefore, regular ethics forums should be a part of organisational structures^46^ enabling shared reflection and lifelong learning for nurses.
